# Oral Astragalus polysaccharide alleviates adenine-induced kidney injury by regulating gut microbiota–short-chain fatty acids–kidney G protein-coupled receptors axis

**DOI:** 10.1080/0886022X.2024.2429693

**Published:** 2024-11-27

**Authors:** Wenbo Liu, Yuanyuan Zhang, Dongmei Hu, Lihua Huang, Xusheng Liu, Zhaoyu Lu

**Affiliations:** a State Key Laboratory of Dampness Syndrome of Chinese Medicine, The Second Clinical College of Guangzhou University of Chinese Medicine, Guangzhou, China; b Nephrology Department, The Second Affiliated Hospital of Guangzhou University of Chinese Medicine, Guangdong Provincial Hospital of Chinese Medicine, Guangzhou, China

**Keywords:** Chronic kidney disease, gut microbiota, Astragalus polysaccharide, short-chain fatty acids, renal protective

## Abstract

Chronic kidney disease (CKD) can cause gut microbiota dysbiosis and decreasing production of short-chain fatty acids (SCFAs), which aggravate the injury of kidney. It has been found that a variety of Chinese medicine polysaccharides can regulate gut microbiota, especially probiotics, and have beneficial effects on human health. Astragalus polysaccharide (APS) is a major component of *Astragalus aceus*. The aim of this study was to investigate whether APS can regulate gut microbiota–SCFAs to slow the progression of CKD. Adenine-induced CKD mice (Ade) were established and APS was treated. The renal protection of APS on CKD mice was evaluated by renal function and pathological staining of renal tissues. Feces samples were collected for 16SrRNA sequence and LC–MS/MS analysis. Kidney G protein-coupled receptor (GPR) levels were also detected in renal tissue. APS supplementation can reduce serum creatinine and urea nitrogen levels in mice model (Ade) and attenuate renal tubular interstitial injury and renal fibrosis. Further application of 16SrRNA sequencing showed that the abundance of SCFA producing bacteria, such as *Kineothrix*, *Faecalibaculum*, *Akkermansia*, *Lactobacillus*, and *Roseburia*, was upregulated after APS treatment. Fecal LC–MS/MS detection showed that the levels of acetate, propionate and butyrate in Ade mice increased after APS supplementation. The detection of renal GPRs showed that APS supplementing could significantly increase the levels of renal GPR41 and GPR43, and also partially increase the levels of GPR109a in Ade mice. Our research confirms that APS supplementation can upregulate the abundance of SCFA producing bacteria and increase SCFA levels to attenuate renal tubular interstitial injury and fibrosis via GPRs.

## 1. Introduction

Chronic kidney disease (CKD) has become a public health problem threatening global human health, and its incidence is increasing year by year. When CKD progresses to end-stage renal disease (ESRD), the prognosis is poor and the treatment cost is very high [1].

Over the past decade, many studies [2,3] have revealed the interaction between the kidney and the colon in CKD, which is known as the CKD – colon axis. On one hand, CKD can lead to gut microbiota imbalance and impaired intestinal barrier function [4]. On the other hand, the disruption of intestinal homeostasis leads to the decrease of probiotics and short-chain fatty acid (SCFA) levels, while the production of uremic toxins significantly increases, and also can lead to the translocation of intestinal bacteria and endotoxins, leading to systemic inflammation and oxidative stress, increasing kidney damage and accelerating the progression of CKD [5,6].

Short-chain fatty acid is a kind of fatty acid with less than six carbon atoms, which is produced by gut microbiota in the fermentation of dietary fiber, mainly including acetate, propionate, butyrate, etc. [7]. The gut microbiota producing SCFAs are mainly *Bifidobacterium*, *Lactobacillus*, and *Clostridium butyricum* [8,9]. The change of SCFAs in patients with CKD is related to the decrease of the abundance of gut SCFA producing bacteria, and the degree of decrease is positively correlated with disease progression [10,11]. SCFA plays a clear role in slowing the progression of CKD, including inhibiting inflammatory response, inhibiting oxidative stress, regulating autophagy, improving energy metabolism and immune pathways [12]. Supplementation with SCFAs (such as acetic acid, propionic acid, or butyric acid), either directly or through dietary fiber or nutritional therapy to regulate the gut microbiota in favor of SCFA producing bacterial species, has a positive impact on the management of CKD [13,14]. A clinical study published when CKD patients were treated with SCFAs observed significant improvements, including weight stabilization; pro-inflammatory factor decreased significantly, while the anti-inflammatory cytokine IL-10 increased significantly [15].

*Astragalus aceus* is a kind of precious Chinese medicine, which is one of the most key drugs in the treatment of CKD in traditional Chinese medicine [16]. Astragalus polysaccharide (APS) is the main component of Astragaloside [17]. Most polysaccharides cannot be directly digested and absorbed by the body. As a non-starch polysaccharide, APSs cannot be fully digested by the stomach and small intestine, thus reaching the gut [18].

At present, it has been found that a variety of traditional Chinese medicine polysaccharides can regulate gut microbiota, especially probiotics, and have beneficial effects on human health [19]. However, the effects of APSs on CKD gut probiotic–SCFAs have not been well described. Therefore, the aim of this study was to determine the effects of APS supplementation on the gut–kidney axis in a mouse model of CKD.

## 2. Materials and methods

### 2.1. Mice and dietary intervention

C57BL/6 mice were provided by Guangdong GemPharmatech Co., Ltd. (Shishan Town, China) and raised in a special pathogen free animal rearing room of Guangdong Hospital of Chinese Medicine at a constant ambient temperature of 24 °C and a day and night cycle of 12 h. Considering that the 5/6 nephrectomy may interfere with the peritoneal and gut microbiota of mice, the adenine feeding method was used to establish a CKD mouse model. The specific implementation process was to add 0.2% adenine to the mouse diet, that is, the mice were fed 0.2% adenine diet for 6 weeks [20]. Adenine feed is manufactured by Guangdong Medical Laboratory Animal Center (Guangzhou, China). This study has been approved by the Institutional Ethics Review Committee of Guangdong Provincial Hospital of Chinese Medicine (No. 2022057).

### 2.2. Astragalus polysaccharide treatment

Astragalus polysaccharide (purity >90%) is provided by Beijing Solaibao Technology Co., Ltd. (Beijing, China). Mice fed 0.2% adenine were simultaneously given oral APS at a daily dose of 200 mg/kg or 400 mg/kg for 6 weeks. Control adenine feed mice and normal feed mice were given the same amount of pure water orally every day. So, this study was divided into four groups: normal group, adenine group, APS-L, and APS-H treatment group, *n* = 8 in each group. Weekly measurements of body weight and food intake were recorded. When calculating the food intake of mice, enough feed is placed in the mouse cage and weighed, and the remaining feed is weighed after seven days. The amount of feed consumed/days/number of mice per cage is the food intake of the mice.

### 2.3. Histology

After the mice were sacrificed under excessive anesthesia, kidney specimens were taken, fixed with 4% paraformaldehyde (PFA), embedded in paraffin, sliced to 3 μm, and stained by hematoxylin and eosin (H&E) and Masson staining. Abnormal parenchyma is considered to be present in one or more of the following conditions: tubule inflammation, tubule dilation or disappearance, tubule atrophy, interstitial inflammation and fibrosis. The quantitative scoring method is as follows: the extent of abnormal renal parenchyma and fibrotic area were visually estimated as a percentage of the total area in well-oriented sections which included both the renal cortex and medulla [20].

### 2.4. Serum creatinine, urea nitrogen, and albumin detection

Serum creatinine (Scr), urea nitrogen, and albumin were determined by creatinine (Cr), urea, and albumin assay kit (Nanjing Jiancheng Bioengineering Institute, Nanjing, China), according to manufacturer’s instructions.

### 2.5. Immunohistochemistry

Paraffin-embedded colon sections were dewaxed, rehydrated, and immersed in 3% hydrogen peroxide for 10 min at room temperature to block endogenous peroxidase activity, followed by antigen retrieval for 15 min. All sections were blocked with 5% blocking buffer for 30 min. Sections were incubated with anti-G protein-coupled receptor 41 (GPR41) primary antibody (1:100, Lot: 2440.Pb1.AP, NOVUS, Centennial, CO), anti-GPR43 primary antibody (1:50, Lot: #617A024, Absin, Shanghai, China), and anti-GPR109a primary antibody (1:100, Lot: E105361, NOVUS, Centennial, CO) at 4 °C overnight. The next day sections were washed and incubated with species-specific secondary antibody (Boster, Wuhan, China) and then developed with 3,3′-diaminobenzine (DAB, Boster, Wuhan, China) and counterstained with hematoxylin.

### 2.6. Western blot analysis

Kidney protein was separated by SDS-PAGE electrophoresis and electroimprinted on polyvinylidene fluoride (PVDF) membranes. The membranes were blocked with QuickBlock™ Blocking Buffer (Beyotime, Shanghai, China), then incubated with a primary antibody, anti-GPR41 (1:1000, NOVUS, Centennial, CO), GPR43 (1:500, Absin, Shanghai, China), GPR109a (1:1000, NOVUS, Centennial, CO), or GAPDH (1:3000, Lot: #8, Cell Signaling Technologies, Boston, MA) overnight at 4 °C. After thorough cleaning with TBS-T solution for three times, incubation with HRP-conjugated secondary antibody (1:2000, Cell Signaling Technologies, Boston, MA) was performed. The immune response bands were visualized using chemiluminescent HRP substrate (Millipore, Billerica, MA) and Bio-Rad ChemiDoc XRS+ gel imaging system (Hercules, CA).

### 2.7. Processing of 16SrRNA sequences and bioinformatics and biostatistics

Fecal DNA samples were extracted from mice using NucleoSpin Soil kit-Macherey-Nagel (Biocompare, Freiburg, Germany). The PCR reaction system was configured with 30 ng qualified genomic DNA samples and corresponding fusion primers, and the PCR reaction parameters were set for PCR amplification. Agencourt AMPure XP magnetic beads were used to purify the PCR amplification products and dissolve them in elution buffer. Labels were attached to complete the library construction. The fragment range and concentration of the library were measured using Agilent 2100 Bioanalyzer (Santa Clara, CA). The qualified libraries were sequenced by MGI2000 sequencer.

Disembarkation data filtering, remaining high-quality clean data for later analysis. Reads were spliced into Tags according to the overlap between reads. FLASH software was used for sequence splicing (Fast Length Adjustment of Short reads, v1.2.11). Cluster Tags into operational taxonomic units (OTUs), compare with databases, and annotate species. Based on OTU and annotation results, sample species complexity analysis, inter-group species difference analysis, and association analysis were performed.

### 2.8. Short-chain fatty acid detection

Fecal SCFA levels of mice were detected by LC–MS/MS using MRM scanning mode. First, the samples were processed, the mouse feces were mixed with methanol–acetonitrile mixture and magnetic beads, and then centrifugated for use. Standard curve preparation: seven kinds of SCFAs were mixed and gradient dilution was performed. Fecal suspension samples or standard kojic samples were added with MeOH/ACN, shockingly precipitated, and then 3-NPH and EDC-6% pyridine mixture were added and incubated in a metal both at 40 °C. Add 1000 D of internal standard solution. The liquid after centrifugal filtration was fed on LC–MS.

The testing platform of LC–MS/MS was the Waters Iclass-AB Sciex 6500 liquid mass spectrometry system (Milford, MA). The parameters of the liquid chromatography were as follows: Waters BEH C18 column (model: 1.7 μm × 2.1 × 100 mm), column temperature: 40 °C, mobile phase: phase A: H_2_O + 0.1% FA, B phase: ACN + 0.1% FA, sample volume 10 μL. Mass spectrum parameters: ion source: ESI detection mode: negative ion mode, ion source temperature: 450 °C, and detector voltage: −4200 V.

Finally, the SCFA concentration was calculated. The default parameters were used in MultiQuant software (SCIEX, Framingham, MA) to automatically identify and integrate each MRM transition (ion pair), and the SCFA content of SCFAs was calculated with the aid of manual inspection.

### 2.9. Statistical method

SPSS software (Version 22.0, IBM Corp., Armonk, NY, USA) was used for statistical analysis. We first conducted a normality test, and the data results that met the normal distribution were expressed as mean ± standard deviation (*x* ± *s*). Univariate ANOVA was used to compare the mean values of multiple groups of independent measurement data meeting normal distribution. Tukey’s test was used to compare and analyze differences between groups when variances were homogeneous. Dunnett *T*3 test was used to compare and analyze differences when variances were heterogeneous. *p* Value <.05 indicates statistical significance (*) and *p* value <.01 indicates high statistical significance (**).

## 3. Results

### 3.1. APS improves kidney function in adenine-induced CKD mice

**Figure 1.**
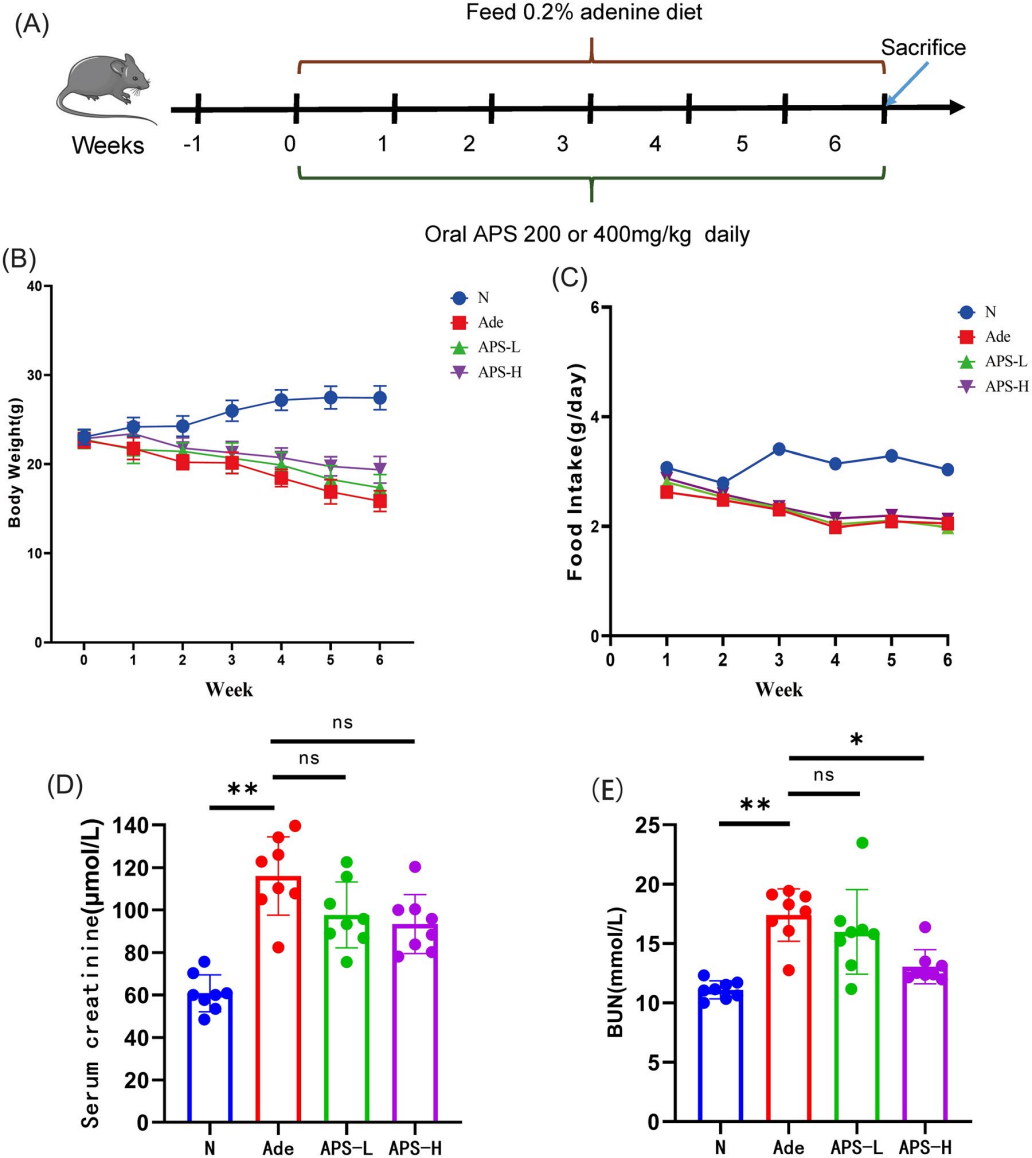
Effects of APS on adenine-induced CKD mice. (A) Experimental design. (B) Body weight. (C) Food intake. (D) Serum creatinine. (E) Blood urea nitrogen. The data are presented as the mean ± standard deviation of the mean for each measurement (*p < .05, **p < .01).

The complete experimental design is shown in Figure 1(A). Compared with the normal group, adenine-induced (Ade) mice began to show significant weight loss and food intake decrease after starting 0.2% adenine diet for one week, and lasted for 6 weeks (*p* < .01, respectively, Figure 1(B,C)). Compared with the Ade group, the body weight of mice in the APS low and high dose intervention groups increased slightly, and there was no significant difference in food intake (Figure 1(B,C)). Serum creatinine and blood urea nitrogen (BUN) levels of Ade mice were significantly higher than those of normal diet mice (*p* < .01, respectively, Figure 1(D,E)). The serum urea nitrogen level of Ade mice supplemented with high-dose APS was significantly lower than that of untreated Ade mice (*p* < .05), and the Scr level was also decreased to some extent (Figure 1(D,E)). High-dose APS improved renal function in Ade mice better than low-dose APS (Figure 1(D,E)).

### 3.2. APS supplementation alleviates kidney injury in Ade mice

**Figure 2.**
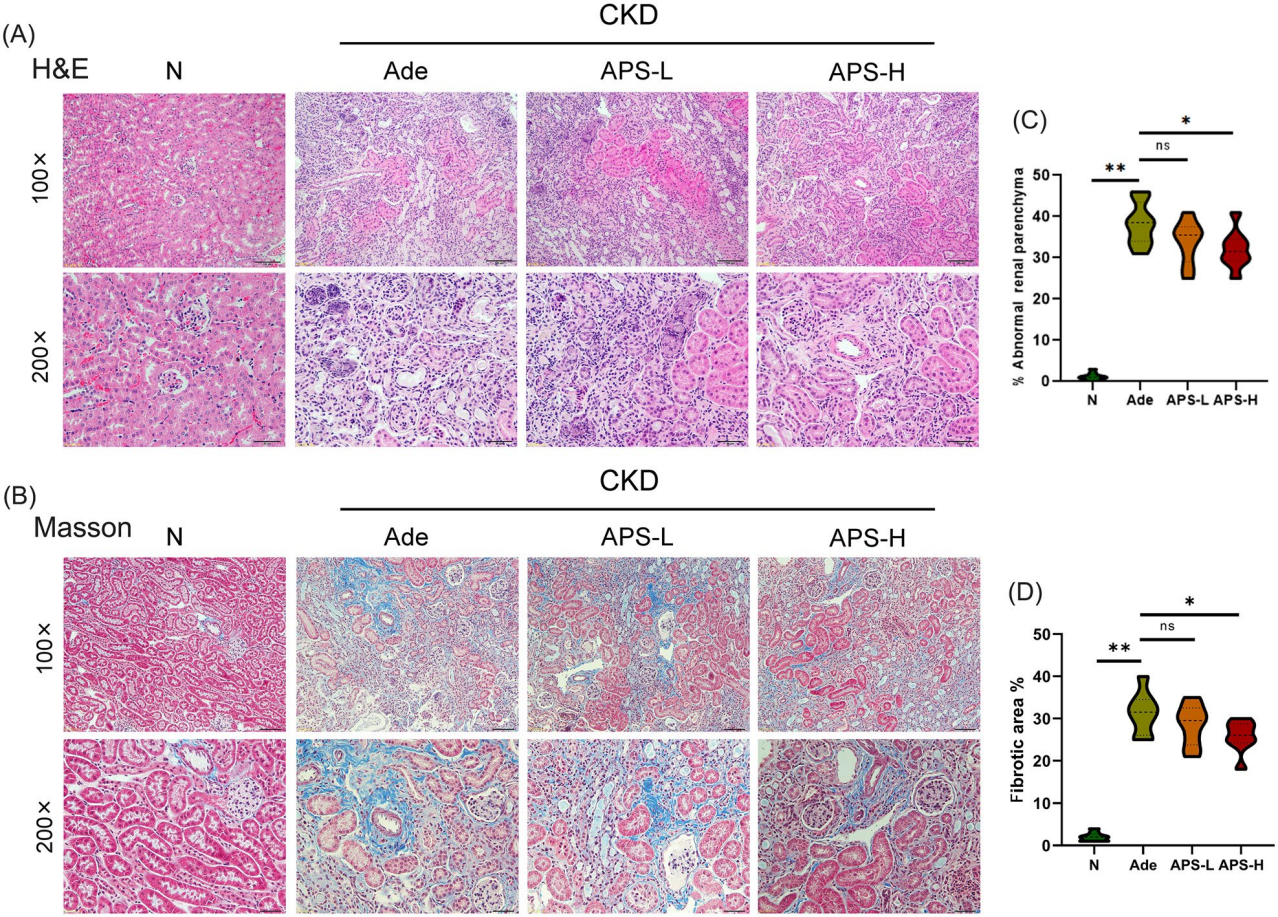
APS alleviates kidney injury and fibrosis in Ade mice. (A) H&E staining of kidney tissue (×100 and ×200). (B) Masson staining of kidney tissue (×100 and ×200). (C) The extent of abnormal renal parenchyma by H&E staining. (D) Area of kidney fibrosis by Masson staining. The data are presented as the mean ± standard deviation (*p < .05, **p < .01).

In order to determine the therapeutic effect of APS on Ade mice, we performed H&E and Masson staining on paraffin sections of kidney tissue to examine the pathological changes of kidney in each group. No damage was found in renal tubule interstitium in normal diet mice. Untreated Ade mice showed significant renal tubule atrophy, lumen dilation, interstitial monocyte infiltration, and interstitial fibrosis (Figure 2(A,B)), and Ade mice had significantly higher percentage abnormal renal parenchyma and renal fibrosis area than normal mice (*p* < .01, respectively, Figure 2(C,D)). After high dose APS treatment, the renal pathological injury of CKD mice was significantly improved, and the percentage abnormal renal parenchyma and renal fibrosis area were lower than those of untreated Ade mice (*p* < .05, respectively, Figure 2(C,D)). The therapeutic effect of APS-H dose was superior to APS-L (Figure 2), suggesting that supplementing APS can improve the progression of kidney injury in Ade mice.

### 3.3. Changes of gut microbiota after APS treatment

**Figure 3.**
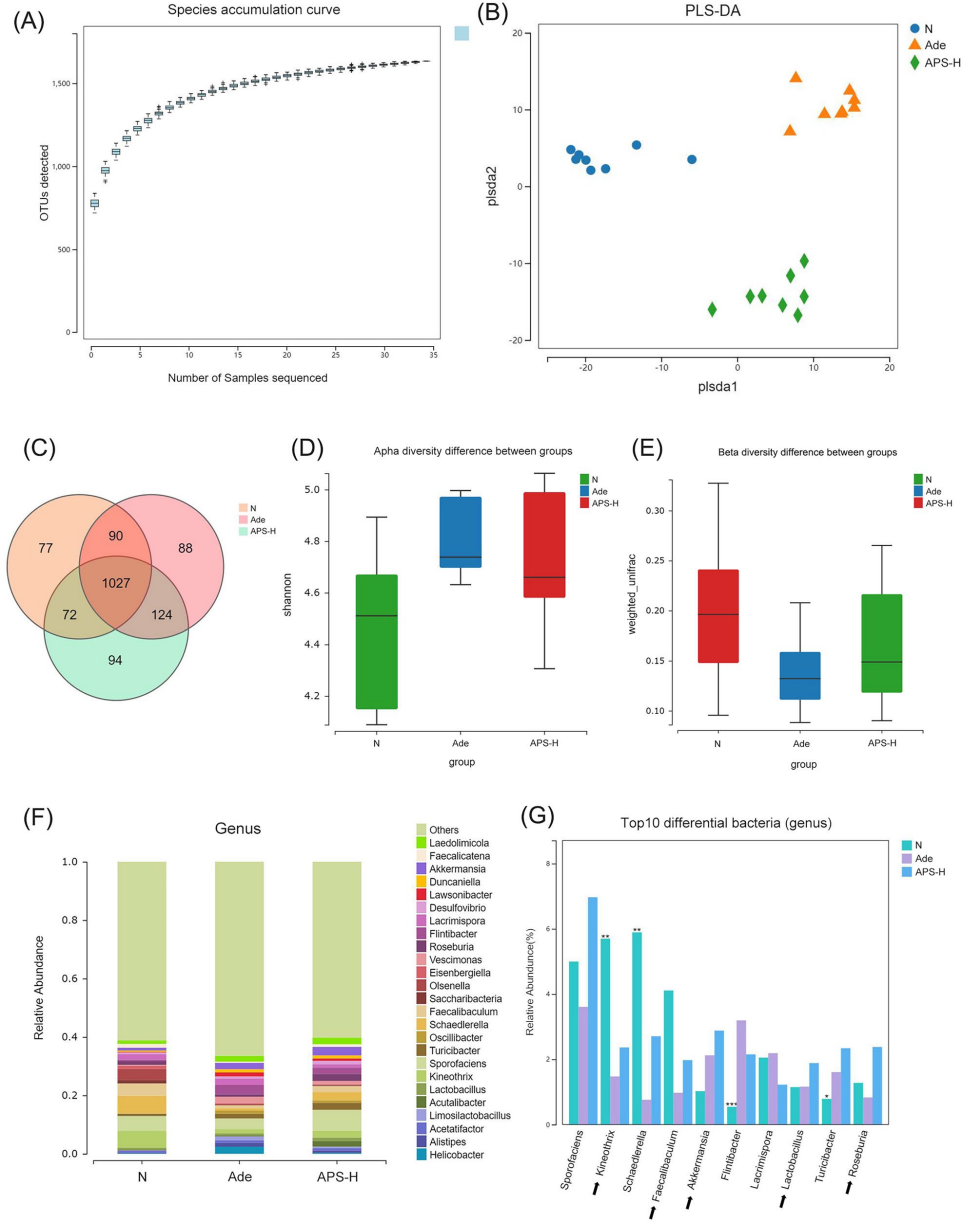
Regulation of APS on gut microbiome in Ade mice. (A) Species accumulation curve. (B) Partial least squares discrimination analysis (PLS-DA) to compare *β*-diversity of gut microbiota. (C) Venn diagram of OTUs. (D) The Shannon analysis of α-diversity. (E) The weighted UniFrac analysis of *β*-diversity. (F) Compositions of gut microbiota at the genus level. (G) Top 10 different bacteria among the three groups in genus level.

We selected normal, Ade, and APS-H groups for fecal gut microbiota analysis, using 16SrRNA sequencing. The species accumulation curve is an effective tool for understanding community composition and predicting species richness. It can be used to assess sample adequacy and estimate species richness. In this study, the slope at the end of the species accumulation curve tends to flatten out, indicating an adequate sampling rate (Figure 3(A)).

PLS-DA visually showed that the microbiome structure in the Ade group was significantly different from that in the normal and APS-H treated groups (Figure 3(B)). Venn diagram shows that the common number of OTUs among normal, Ade, and APS-H groups is 1027, and each group has about 80 different OTUs (Figure 3(C)). α-Diversity analysis showed that the Shannon index of Ade group was higher than that of normal group, and that of APS-H intervention group was lower than Ade group (Figure 3(D)). Weighted UniFrac *β*-diversity analysis showed that gut microbiota in Ade group had significant changes compared with normal group, and the gut microbiota in APS-H group was more similar to that in normal group (Figure 3(E)). Analysis of the difference between groups showed that APS-H had a significant regulatory effect on the gut microbiota of Ade mice (Figure 3(F)). At the genus level, *Sporofaciens*, *Kineothrix*, *Schaedlerella*, *Faecalibaculum*, *Akkermansia*, *Flintibacter*, *Lacrimispora*, *Lactobacillus*, *Turicibacter*, and *Roseburia* are the top 10 different among the three groups (Figure 3(G)). Compared with Ade group, after APS-H treatment, *Sporofaciens*, *Kineothrix*, *Schaedlerella*, *Faecalibaculum*, *Akkermansia*, *Lactobacillus*, *Turicibacter*, and *Roseburia* increased in relative abundance. The relative abundance of *Flintibacter* and *Lacrimispora* showed a decreasing trend (Figure 3(F,G)).

**Figure 4.**
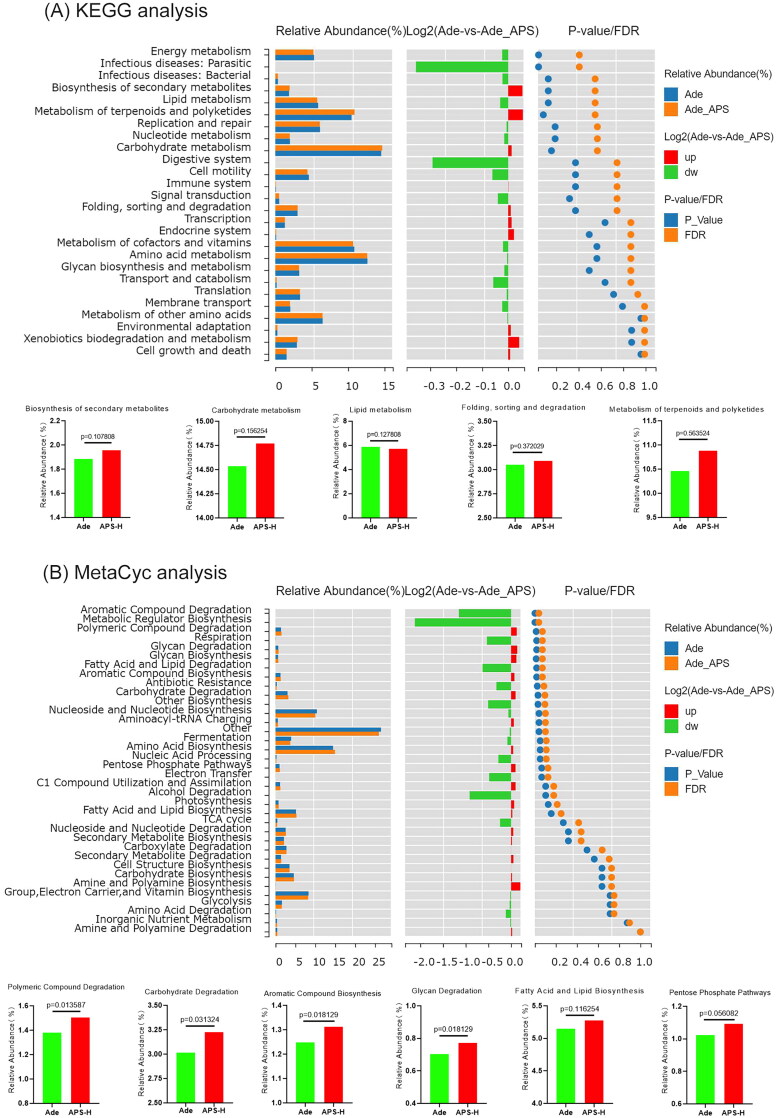
Functional analysis of gut microbiota. (A) The functional differences between Ade group and APS-H group by KEGG functional analysis. (B) MetaCyc function analysis between Ade group and APS-H group by PICRUST2.

Kyoto Encyclopedia of Genes and Genomes (KEGG) functional analysis showed that the functional differences between Ade group and APS-H group are mainly concentrated in secondary metabolite biosynthesis, lipid metabolism, carbohydrate metabolism, folding, sorting and degradation, metabolism of terpenoids and polyketides (Figure 4(A)). PICRUST2 was used to predict the abundance of MetaCyc pathway in bacterial communities. MetaCyc function analysis suggested that the differences in metabolic function between Ade and APS-H groups were mainly due to polymeric compound degradation and glycan degradation, aromatic compound biosynthesis, carbohydrate degradation, pentose phosphate pathways and fatty acid and lipid biosynthesis (Figure 4(B)).

In conclusion, APS supplementation can affect the abundance and metabolism of intestinal flora in Ade mice through polysaccharide degradation, carbohydrate utilization, fatty acid and lipid biosynthesis and secondary metabolite biosynthesis. Among them, *Kineothrix*, *Faecalibaculum*, *Akkermansia*, *Lactobacillus*, and *Roseburia* are known as SCFA producing bacteria. The abundance of *Kineothrix*, *Faecalibaculum*, *Akkermansia*, *Lactobacillus*, and *Roseburia* in mice treated with APS was up-regulated, and APS may reduce kidney injury by up-regulating SCFAs.

### 3.4. Upregulating SCFAs by APS treatment

In order to evaluate whether APS can enhance the level of SCFAs by regulating the gut microbiota of CKD mice, especially the SCFA producing bacteria, we further measured the level of SCFAs in mice feces.

**Figure 5.**
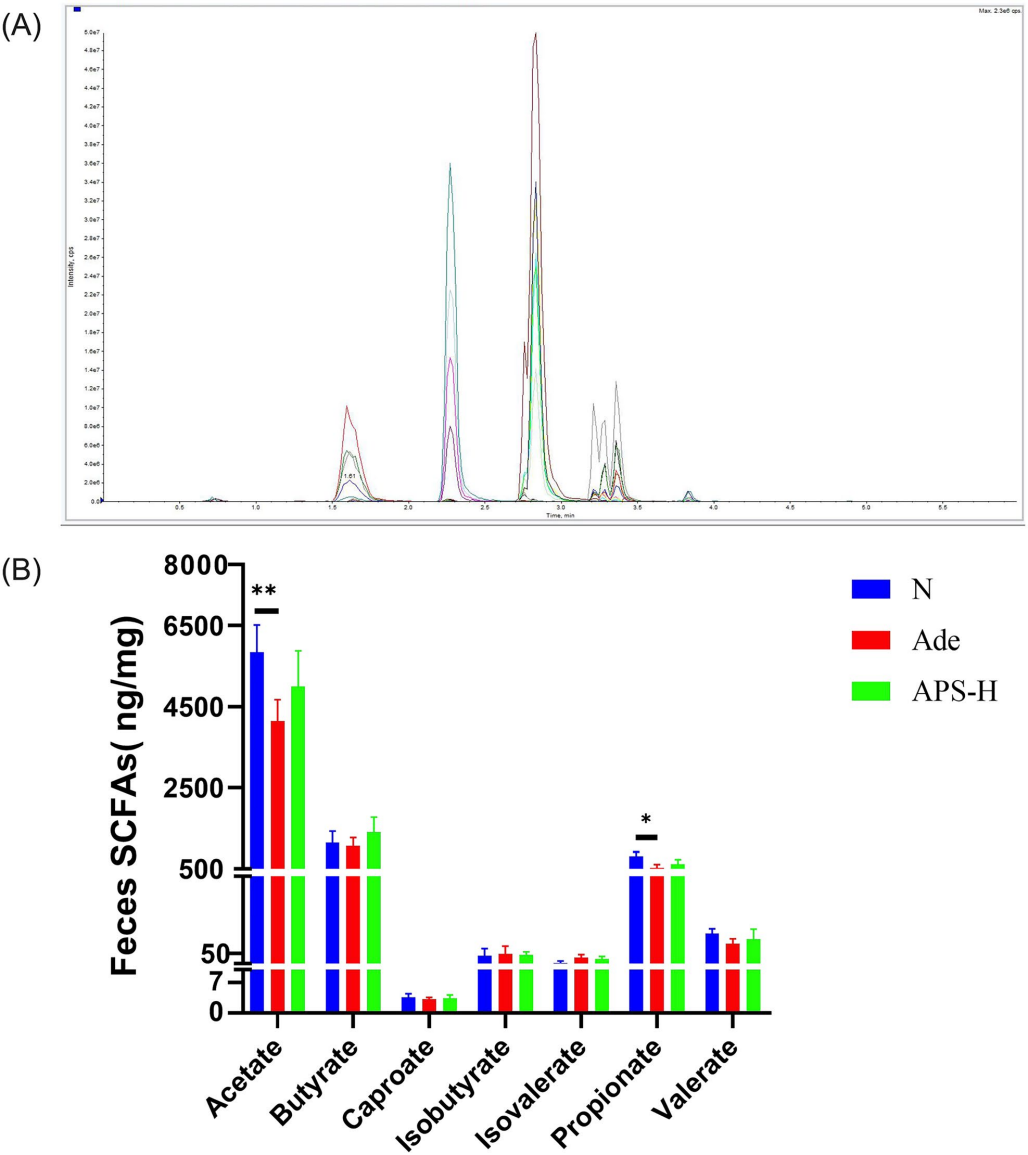
Upregulating SCFAs by APS treatment in Ade mice. (A) Extraction ion flow diagram of QC samples in LC–MS/MS analysis. (B) The level of feces SCFAs. Data are presented as the mean ± standard deviation (*p < .05, **p < .01).

The extraction ion flow diagram (Figure 5(A)) of QC samples indicated that the quality of this LC–MS/MS test was qualified. LC–MS/MS analysis showed that acetate, propionate, and butyrate were indeed the most abundant SCFAs in gut, accounting for about 95% of the total, of which acetic acid is the most abundant. Compared with the normal group, the acetate, propionate, and butyrate of Ade mice were significantly decreased. Ade mice feces acetate, propionate, and butyrate increased to a certain extent after APS-H treatment (Figure 5(B)).

### 3.5. APS supplementing increase kidney GRPs expression

**Figure 6.**
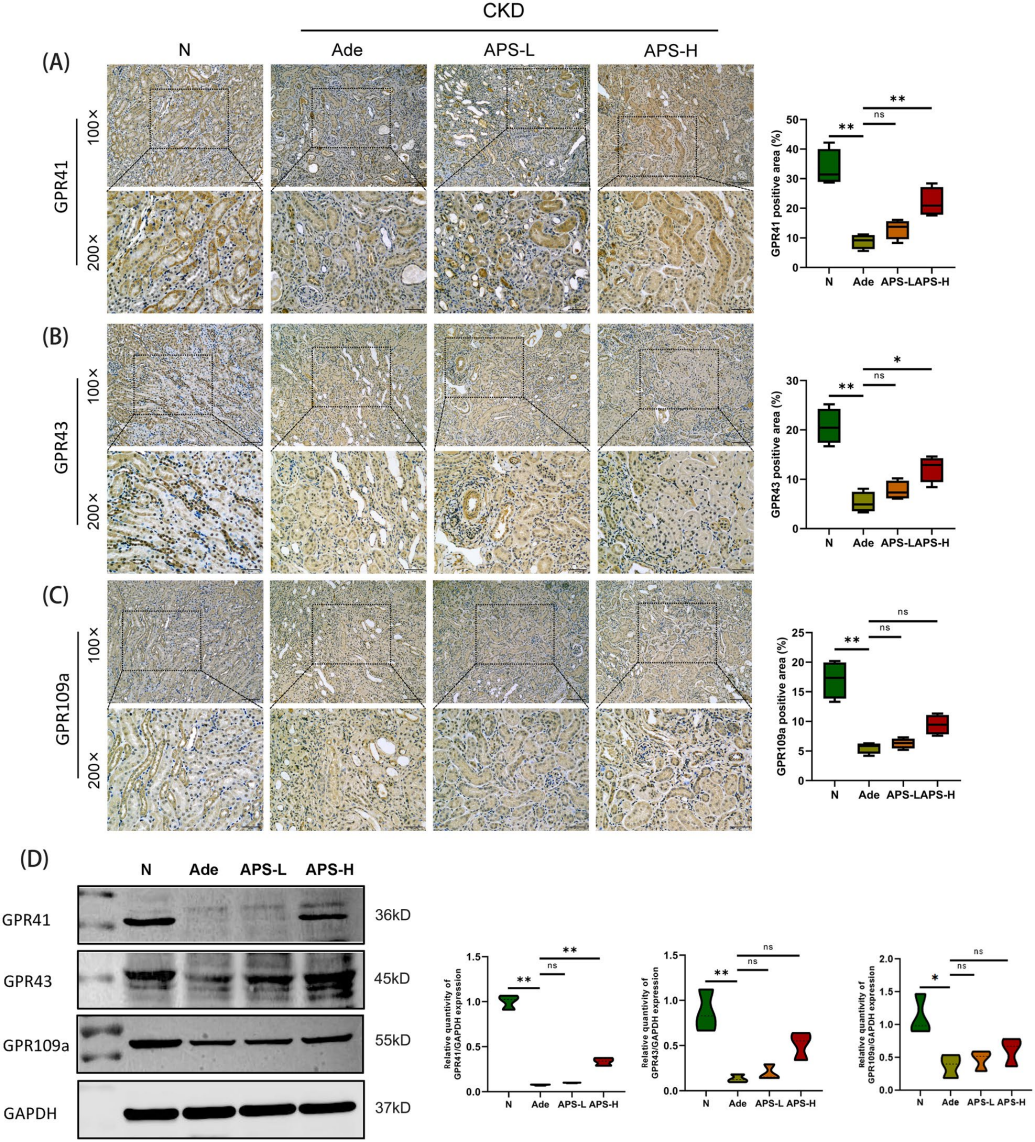
APS supplementing increase kidney GRP expression. (A) Kidney GPR41 immunohistochemical staining and analysis (×100 and ×200). (B) Kidney GPR43 immunohistochemical staining and analysis (×100 and ×200). (C) Kidney GPR109a immunohistochemical staining and analysis (×100 and ×200). (D) Western-blot analysis of GPR41, 43, and 109a in kidney tissue. The data are presented as the mean ± standard deviation of the mean for each measurement (*p < .05, **p < .01).

The expressions of SCFA key receptors GPR41, GPR43, and GPR109a in kidney were detected by Western blot analysis and immunohistochemistry. Western blot analysis and immunohistochemistry results indicated that GPR41, GPR43, and GPR109a were abundant in the normal group (Figure 6(A–D)), and GPR41 was mainly expressed in proximal tubule cells (Figure 6(A)), and GPR43 and 109a were mainly expressed in distal tubules and collecting tubules (Figure 6(B,C)). Both immunohistochemical and Western blot analysis results indicated that renal GPR41, 43, and 109a expressions were significantly down-regulated in Ade group (*p* < .01, respectively, Figure 6(A–D)). APS-H supplementing could significantly increase the levels of renal GPR41 and GPR43, and also partially increase the levels of GPR109a in Ade mice (Figure 6(A–D)). It is worth noting that both immunohistochemical staining and Western blot analysis results showed that Ade mice kidney GPR41 significantly reversed after APS-H supplementation (*p* < .01, respectively).

## 4. Discussion

The human gut is home to millions of trillions of microbes, known as the human ‘second genome.’ More and more studies have found that the progression of CKD is related to intestinal microecological disorders [21], and it has been proposed that there is a close connection between the gut and kidney, which is called the ‘chronic kidney disease – colon axis’ [2,3]. The metabolites of intestinal flora are the important mediators connecting the gut and kidney. Short-chain fatty acids are the main metabolites of gut microbiota, which enter the blood circulation as signaling molecules and act on peripheral tissues to play biological roles [22].

SCFAs are saturated fatty acids with chain length of 1–6 carbon atoms and are the main products of dietary fiber fermentation in the colon, among which acetate, propionate, and butyrate are the most abundant SCFAs in the human body, accounting for about 95% of the total [23]. Acetate is mainly produced by intestinal fermentation of *Bacteroides*, *Bifidobacterium*, *Akkermansia*, *Prevotella*, *Ruminococcus*, and *Clostridium*. Propionate is mainly produced by *Clostridium*, *Ruminococcus*, and *Roseburia*. Butyrate is mainly produced by the fermentation of *Eubacterium*, *Faecalibacterium*, *Coprococcus*, *Roseburia*, and *Clostridium* [7].

Existing data support a link between altered gut microbiome and human kidney disease, particularly with respect to the contraction of SCFA producing bacteria [10,11]. Compared with the gut microbiota of healthy people, there are differences in the relative abundance of about 190 types of bacteria in CKD patients [24], mainly manifested as a decrease in the relative abundance of SCFA producing bacteria, including *Ruminococcus*, *Eubacterium*, *Clostridium*, and *Roseburia* [10,25,26]. Other studies have found a decline in the relative abundance of Prevotellaceae in CKD patients, which contain two key enzymes for butyric acid formation: phosphotransbutyrylase and butyrate kinase [27]. Meanwhile, serum SCFA level in CKD patients is low, and butyrate level is negatively correlated with renal function [11].

The kidney and gut are closely linked, and intestinal dysbiosis can affect kidney function. SCFA is produced by the symbiotic gut microbiota and affects the kidney through multiple mechanisms. Including interaction with homologous receptors in kidney cells, it exerts anti-inflammatory and oxidative stress effects to delay CKD progression by inhibiting the NF-κB and MAPK pathways [12,28]. The expression of GPR41, GPR43, and GPR109a has been reported in kidney tissue [29]. After renal ischemia–reperfusion injury, the expression of GPR41 and GPR43 is decreased, and acetate therapy can restore the expression of GPR43, improve inflammation and renal dysfunction. Similar renal protection was observed in ischemic animals treated with acetate, propionate, and butyrate producing bacteria [30]. Conversely, GPR43- or GPR109a-deficient mice were not protected by the SCFAs, suggesting that renoprotection was dependent on these receptors [31].

Therefore, nutritional strategies aimed at increasing SCFAs may be beneficial for patients with CKD, and a single-center, non-randomized study showed that SCFA supplementation reduced C-reactive protein, IL-2 and IL-17, oxidative stress, enteric-derived indoxyl sulfate, and p-cresol sulfate in patients with maintenance hemodialysis [14]. Other study has found that SCFA supplementation can reduce systemic inflammation in ESKD patients, and this effect is related to the expansion of circulating Tregs [32]. Treatment associated with SCFAs can be a therapeutic strategy for CKD.

Traditional Chinese herbal medications (TCHMs) are frequently used in conjunction with western pharmacotherapy for treatment of CKD in China and many other Asian countries. Traditional Chinese medicine works mainly through gastrointestinal tract digestion, metabolism, and absorption to achieve the therapeutic effect. Polysaccharide is one of the main components of traditional Chinese medicine [33], which has anti-inflammatory, antioxidant, immune regulation, glycolipid regulation, and other medicinal activities [34]. More importantly, the safety of plant-derived polysaccharides has been generally recognized compared to other plant metabolites [35]. As non-starch polysaccharides, most plant-derived polysaccharides cannot be absorbed and digested by the upper digestive tract. Instead, they can act as substrates for specific gut bacteria to promote the proliferation of beneficial bacteria, suggesting potential prebiotic activity [36]. In general, intestinal bacteria use polysaccharides as a nutrient source for their proliferative and metabolic activities [37]. A large number of studies have confirmed that polysaccharide can increase the relative abundance of Bacteroidetes, Firmicutes, Lactobacillus, bifidobacterium, and other beneficial bacteria in the gut and reduce the relative abundance of harmful bacteria such as Proteobacteria, Enterococcus, and Fusobacterium nucleatum [19].

To study the interaction between plant-derived polysaccharides and gut microbiota, and to make polysaccharides exert ‘prebiotic effect’ as the target of gut microbiota to intervene in related diseases has a broad application prospect. Polysaccharides are widely found in Chinese medicines with kidney protection, such as Astragalus, Dendrobium, Poria, wolfberry, and *Ganoderma lucidum*, among which Astragalus is one of the most critical drugs in TCM treatment of CKD [16].

In our study, APS supplementation decreased Scr and urea nitrogen levels in adenine-fed mice and ameliorated renal tubular interstitial injury and renal fibrosis. Further application of 16SrRNA sequencing showed that compared to Ade mice. The relative abundance of *Sporofaciens*, *Kineothrix*, *Schaedlerella*, *Faecalibaculum*, *Akkermansia*, *Lactobacillus*, *Turicibacter*, and *Roseburia* increased after APS treatment. The relative abundance of Flintibacter and Lacrimispora showed a decreasing trend. MetaCyc function analysis suggested that the differences in metabolic function between Ade and APS groups were mainly due to polymeric compound degradation and glycan degradation, aromatic compound biosynthesis, carbohydrate degradation and fatty acid and lipid biosynthesis.

The abundance of SCFA producing bacteria such as *Kineothrix*, *Faecalibaculum*, *Akkermansia*, *Lactobacillus*, and *Roseburia* was up-regulated after APS treatment. The results of fecal LC–MS/MS detection showed that the levels of acetate, propionate, and butyrate in Ade mice were significantly increased after APS supplementation. Further detection of renal SCFA receptor GPRs showed that APS supplementing could significantly increase the levels of renal GPR41 and GPR43, especially GPR41, and also partially increase the levels of GPR109a in Ade mice.

Our work shows that APS supplementation can upregulate the abundance of SCFA producing bacteria and increase SCFA levels to attenuate renal tubular interstitial injury and fibrosis via kidney GPRs. We provide a novel strategy for how host–APS–gut microbiota–SCFAs interactions delay CKD progression.

## 5. Conclusions

This article presents new concepts linking APS and gut microbiota–SCFAs–GPRs in CKD, and provides insight into how this may influence future clinical practice and ultimately lead to a paradigm shift in the focus of Chinese medicine polysaccharide recommendations in CKD, particularly with regard to APS.

## Supplementary Material

full uncropped Gels and Blots image.tif

## Data Availability

The datasets used and/or analyzed in this study are available from the corresponding author upon reasonable request. The 16SrRNA sequencing data have been uploaded to the NCBI Sequence Read Archive (SRA): SUB14620968.
