# Supplementary figures and images for: Oral Astragalus polysaccharide alleviates adenine-induced kidney injury by regulating gut microbiota–short-chain fatty acids–kidney G protein-coupled receptors axis
Source: Ren Fail. 2024 Nov 27;46(2):2429693. doi: 10.1080/0886022X.2024.2429693 (PMC11610254; doi:10.1080/0886022X.2024.2429693)

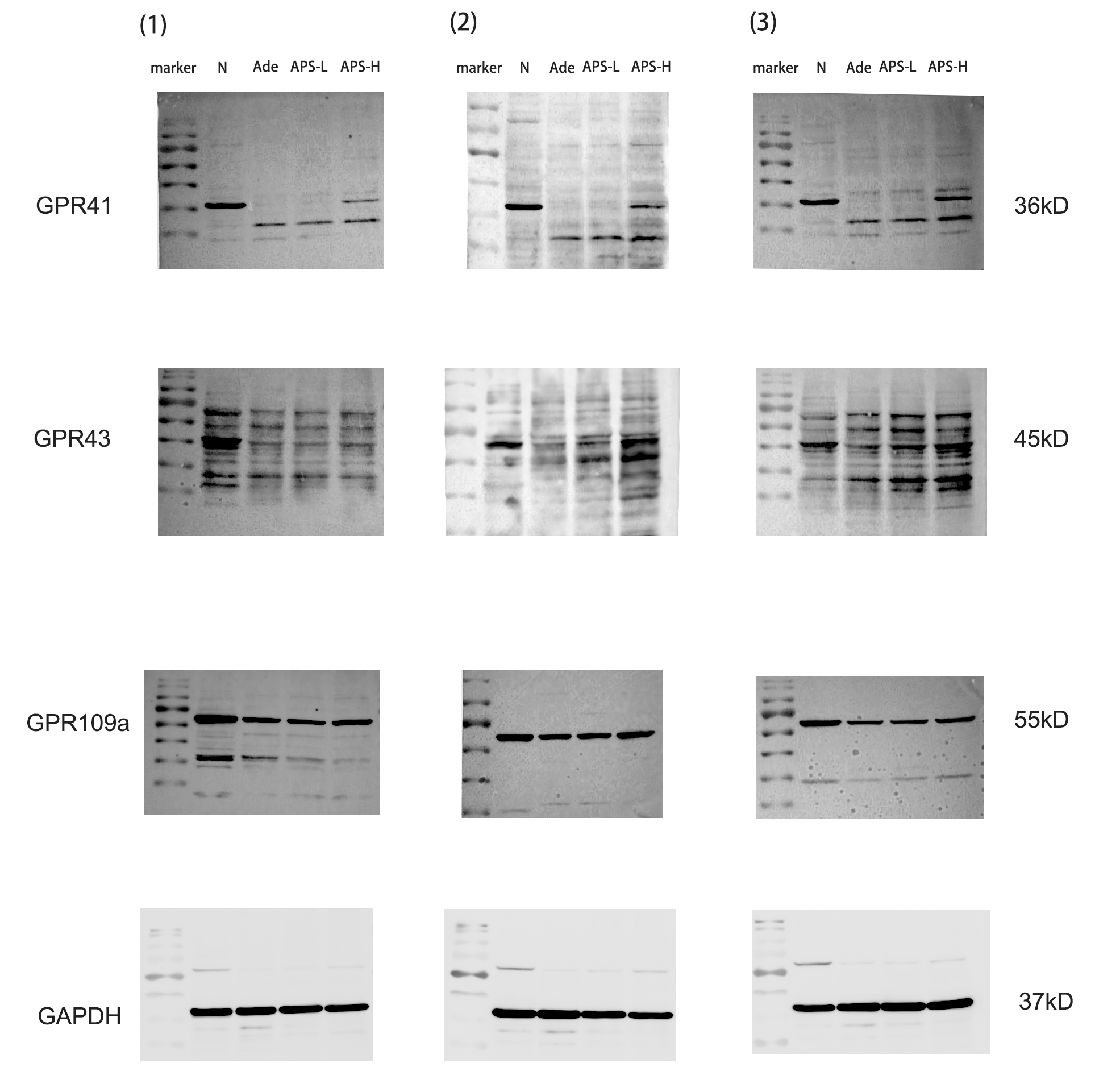

Supplement: full uncropped Gels and Blots image.tif [file IRNF_A_2429693_SM7871.tif]
